# Food for the soul and food for the body. Studying dietary patterns and funerary meals in the Western Roman Empire: An anthropological and archaeozoological approach

**DOI:** 10.1371/journal.pone.0271296

**Published:** 2022-08-24

**Authors:** Domingo C. Salazar-García, Lídia Colominas, Xabier Jordana

**Affiliations:** 1 Departament de Prehistòria, Arqueologia i Història Antiga, Universitat de València, València, Spain; 2 Department of Geological Sciences, University of Cape Town, Cape Town, South Africa; 3 Institut Catala d’Arqueologia Clàssica, Tarragona, Spain; 4 The Tissue Repair and Regeneration Laboratory (TR2Lab), Experimental Sciences and Methodology Department, Faculty of Health Science and Welfare, University of Vic–Central University of Catalonia, Vic, Spain; 5 Research Group of Biological Anthropology (GREAB), Biological Anthropology Unit, BABVE Department, Universitat Autònoma de Barcelona, Cerdanyola del Vallès, Barcelona, Spain; University of Padova: Universita degli Studi di Padova, ITALY

## Abstract

Ancient written sources show that Roman funerary rituals were relevant along the entire Roman Republic and Empire, as they ensured the protection of deities and the memory of the deceased. Part of these rituals consisted of funerary offerings and banquets that were held on the day of the burial, in festivities and other stipulated days. The faunal remains recovered inside the graves and around them are evidence of these rituals. Therefore, their study can allow us to know if the funerary meals and rituals developed in the Roman necropolis were special and implied food that differed from everyday dietary habits, according to the importance of these rituals. To test this, we analysed the archaeozoological and anthropological material from the necropolis of Vila de Madrid (Barcelona, Catalonia), which was in use between the first half of the 2^nd^ century AD and mid 3^rd^ century AD. The archaeozoological analysis of the faunal remains recovered in the necropolis and inside the graves, as well as carbon and nitrogen stable isotope ratios results on bone collagen from 50 faunal specimens and 41 humans, suggest that, overall, funerary meals in Vila de Madrid necropolis did not imply different food than that consumed during life. Regarding age, sex, offerings and diet, some differences are observed, suggesting that inequalities present in life could have been also present in the funerary rituals.

## 1. Introduction

### 1.1. Ancient Roman funerary rituals and food

The afterlife in Roman religion was the milestone that had to be reached after death upon complying with several funerary rituals. Part of these rituals consisted of funerary offerings, banquets and sacrifices of animals, performed to ensure the protection of deities and the memory of the deceased [1–4]. Written sources (Pliny, Epist. IV, 2; Cicero, De leg. II, 22, 55–57; Tacitus, Ann. VI, 5; Petronius, Satyrion 65) show that only when a pig was sacrificed was a grave legally a grave. They also indicate that on the same day of the funeral, a funerary feast was eaten at the grave in honour of the dead and offerings of food were left at the tomb. There was also the *cena novendialis* eaten at the grave on the ninth day after the funeral. Throughout the year there were also other occasions on which the dead were commemorated by funerary meals eaten at the tomb by their relatives and friends, such as their birthdays or several annual festivities (*Parentalia*, *Lemuria*, *Rosalia*) in which a lamb could also be sacrificed [1, 5]. At all of these banquets, the departed had their share set apart for them.

Therefore, through written sources we imagine that banquets and offerings were an important part of the funerary rituals in antiquity. Archaeology has validated this idea. Graves, whether for inhumation or for cremation, are documented widely to have contained holes or pipes through which food and drink could be poured down directly on to the burial, such as at Colchester (UK) [1], Saint-Cyr-sur-Mer (France) [6], Ostia (necropolis d’Isola Sacra, Italy) [7], Tipasa (Mauritaina) [8] or Carmona (necropolis of Puerta de la Sedía, Spain) [9]. Ceramics, faunal and plant remains from these banquets and offerings have also been thoroughly recovered from the necropolis floors and inside the graves. Some examples are the necropolis of Nimes in France [10], the ‘Mausoleo di Blanda Tortora’ in Pergolo in Italy [11], the Eastern cemetery of London in England [12] or the necropolis of Valentia in Spain [13].

Taking into account the importance of these rituals, with this paper we want to take a step deeper into their research. The aim of this study is to investigate whether the funerary rituals involved special food that differed from the every-day diet, or conversely, if what was consumed as food in everyday life was also used in the mortuary meals and ceremonies. To accomplish this, we selected the *collegia funeraticia* area of the necropolis of Vila de Madrid (Barcelona, Catalonia), in use between the first half of the second century AD and the mid third century AD.

### 1.2. C and N isotopes and dietary reconstructions

Carbon and nitrogen stable isotope analysis applied to archaeology is a well-established technique that is routinely used to obtain information on diet from past populations around the world, and has become a must in any research project aiming to evaluate subsistence patterns in the past, especially from prehistoric and ancient times [14]. In this sense, Antiquity is not an exception, and C and N stable isotope analyses have been carried out in a variety of sites from Roman times [15–21]. In the Iberian Peninsula only a few sites have been studied for diet reconstructions with C and N stable isotope analysis [22, 23].

This type of analysis is based on the underlying rationale that the isotopic composition of food eaten is recorded in the body tissues after a predictable isotope fractionation [24]. Archaeological remains can retain the stable isotope ratios present during life, and therefore provide information about the foods an individual consumed. Bone and teeth have specific stable isotope ratios that reflect their chemical origin and formation: the organic part records carbon and nitrogen isotope ratios linked to protein consumption averaged from a number of years prior to death in bone [25], or from specific life-moments of an individual in dentine [26]. Collagen is the preferred substrate for carbon and nitrogen stable isotope analysis, because it is the only major nitrogen source from skeletal remains [27] and provides robust quality indicators that securely record its isotopic integrity [28, 29]. However, it is necessary to consider when interpreting results that stable isotope ratios from bone collagen reflect only the main dietary protein sources consumed several years prior to death [30] rather than that of the diet as a whole, especially for nitrogen [31].

The relative abundance of the stable carbon isotopes ^13^C and ^12^C (δ^13^C) distinguishes the consumption of C_3_ and C_4_ terrestrial resources [32]. Trees, most plants from temperate and cold climates, and most aquatic plants follow the C_3_ photosynthetic pathway (δ^13^C values from -36‰ to -22‰). Herbaceous plants like millet, sorghum, or sugarcane, in both temperate and warm environments, mostly follow the C_4_ photosynthetic process (δ^13^C values from -19‰ to -6‰) [33–35]. Carbon stable isotope ratios also help to define the input on the diet from terrestrial and marine foods [36], although if freshwater or estuarine fish are involved, then the interpretation of δ^13^C values becomes more complex [37]. The δ^15^N isotope values increase by 3–5‰ per trophic level up the food chain, and are usually used to indicate the position of an organism in the food chain [38]. Even if this quantification is less straightforward than previously thought [39], based on the exact values of the nitrogen ratio it is potentially possible to differentiate between individuals that consumed more animal resources from those who consumed less [40]. Furthermore, the fact that aquatic food chains tend to contain more trophic levels than terrestrial ones helps to discriminate between the consumption of marine or C_4_ terrestrial foods when samples are ^13^C enriched [41].

Environmental, physiological and cultural factors may influence the isotopic signal of the food chain, and must be taken into account. Different environmental settings through time and space show different isotopic backgrounds [42]. The "canopy effect" can affect isotope ratios and may result in significantly lower δ^13^C values for plants from closed terrestrial environments compared to their counterparts from open ones [43, 44], while aridity and soil salinity can result in higher δ^13^C and δ^15^N values [45–47]. Physiology also impacts isotopic values, as ruminants have on average higher δ^15^N values compared to non-ruminants [48] and leguminous plants have lower δ^15^N values than that of other plants [49]. Furthermore, individuals subject to hormonal [50], pathological [51], or nutritional [52, 53] stress may have δ^15^N values that no longer reflect the trophic relationships of the ecosystems in which they live. Cultural factors such as the use of manuring [54], food trade networks or migrations can also affect the isotopic baseline values and thus lead to foods or individuals with different values due to their non-local origin. Although some of these factors cannot be controlled, environmental isotope ratio values for humans should be compared to faunal samples recovered from the same archaeological site and stratigraphic level, when possible, or from nearby contemporary fauna to have a more robust dietary interpretation.

## 2. Materials and methods

**Fig 1:**
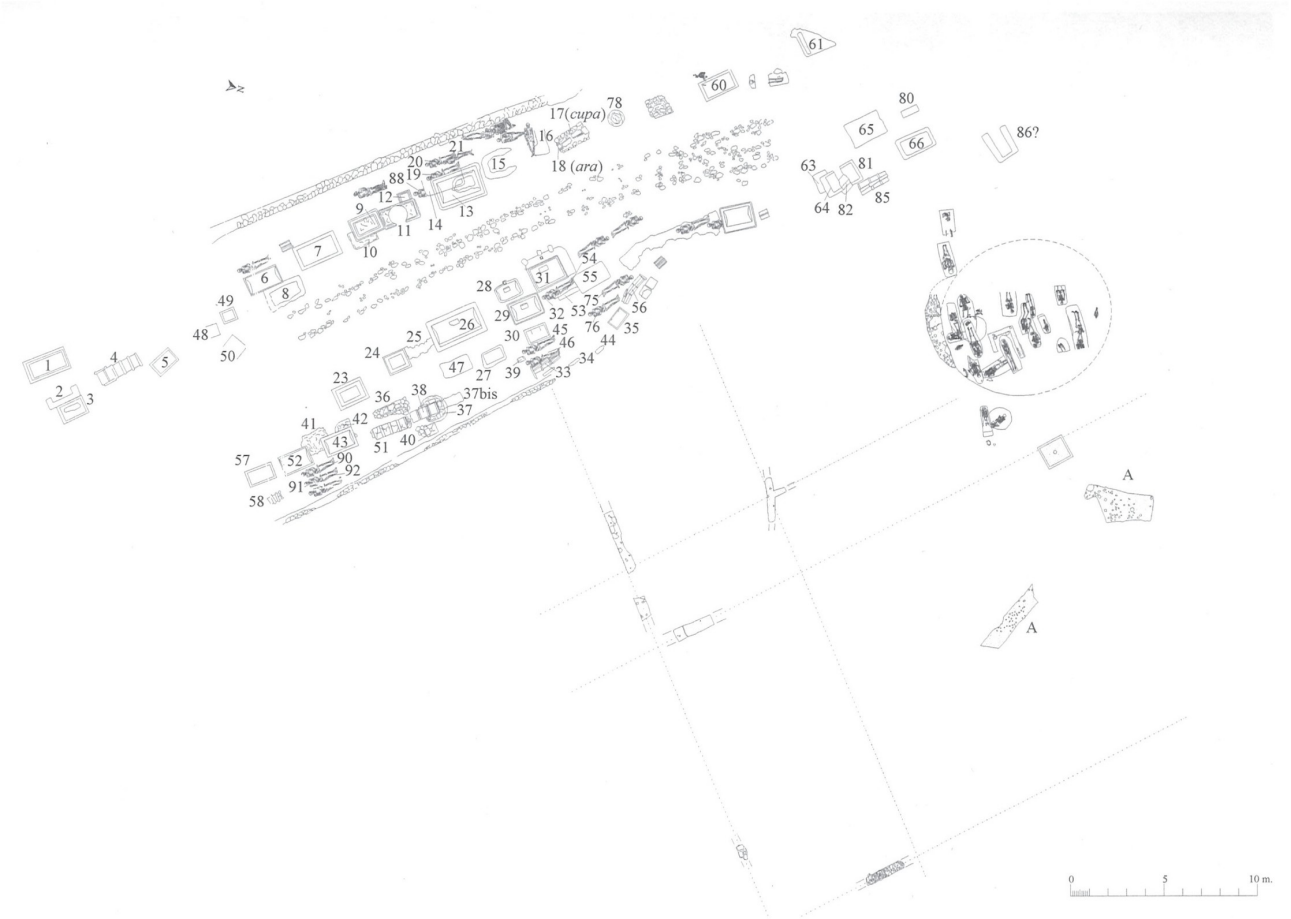
Plan of the Vila de Madrid necropolis with the collective funerary structure located at the north of the secondary road from which the human and animal remains were retrieved (after F. Busquets, I. Pastor. Fondo de excavaciones antiguas-MHCB).

All the materials analysed here were recovered at the ‘Vila de Madrid’ necropolis. The site is located in the city of Barcelona (Catalonia). The first excavations took place in the 1950s. They revealed many burials along a secondary road of what would have been the western necropolis of the Roman city of *Barcino* [55]. Works at the site resumed between 2000 and 2003, during which the presence of a collective funerary structure of about 8.85×5m was noted at the north of the secondary road (Fig 1). This collective funerary structure was interpreted as a *collegium funeraticium*. The *collegia funeraticia* were private associations, sometimes of a professional nature, of free people and slaves of limited purchasing power, who, by paying a monthly fee in life, ensured their burial [55]. A total of 66 burials (59 inhumations and 7 cremations) were recorded within this structure, in use between the first half of the second century AD and the mid third century AD [55]. The materials presented here come from the recent excavations of the collective funerary structure of 2000–2003. The materials were recovered from different contexts of the necropolis. They were recovered from the interior of the inhumations (human and animal remains), from the necropolis’ circulation level (whole carcasses of animals deposited in the necropolis) and from the fill of an abandoned well contemporary to the necropolis occupation phase (animal remains).

### 2.1. Sample selection

Only faunal remains interpreted as waste products from the processing and consumption of meat were selected for the archaeozoological study. This selection was based on the presence of helical fractures, butchery marks and thermo-alterations. Therefore, 342 faunal remains recovered on the occupation floor of the necropolis (NISP = 273) and from the inside of the graves (NISP = 69) were chosen from the whole faunal assemblage, composed of 4882 remains. The other faunal remains were whole carcasses of horses and dogs that were buried in the necropolis, recovered from the circulation level and from inside a well. We selected these 342 faunal remains as indicative of funerary banquets and funerary offerings respectively, considering their location (inside the graves or on the occupation floor of the necropolis) and anthropic modifications (helical fractures, butchery marks and thermo-alterations). Detailed archaeozoological results of the whole assemblage analysis are available [56].

A total of 50 faunal specimens from all contexts of the necropolis (occupation floor, graves and well) were selected for δ^13^C and δ^15^N stable isotope analyses of bone collagen in order to define the local baseline from which to interpret the human values. The selected faunal set was comprised of sheep (n = 4), goats (n = 1), pigs (n = 6), cattle (n = 7), equids (n = 8), dogs (n = 15), foxes (n = 1), rabbits (n = 2), hares (n = 1), chickens (n = 4) and amphibians (n = 1). A total of 41 human individuals representing both sexes and all age ranges present at the site were selected for δ^13^C and δ^15^N stable isotope analyses of bone collagen. Only ribs from primary inhumations were selected. The male/female ratio of the human sample is 1.6:1 (14 males, 9 females, and 18 non-adults of indeterminate sex). The age-at-death of these individuals ranged from post-natal to older than 45 years of age and were grouped in to six age categories: newborn (<1yo), infant I (from 1 to 6 years old), infant II (from 7 to 12 years old), juveniles (from 13 to 18), young adult (from 19 to 30 years old) and adult (more than 30 years of age). The detailed results of the osteological analysis are available in a monograph of the site [57].

### 2.2. Archaeozoological analysis

The osteological analysis was focused on the study of taxonomic and anatomic representation frequencies and age-at-death estimations in order to characterise food funerary preferences. The osteological reference collection from the Universitat Autònoma de Barcelona was used for identification. Sheep and goat differentiation was carried out following a variety of methods [58–60]. The taxonomic variability was based on the relative frequency (NISP) and the Minimum Number of Individuals (MNI). Age-at-death was recorded on the basis of fusion of long bone epiphyses, and of the eruption and wear of mandibular teeth. For cattle and pigs, the tooth wear stages followed the Grant protocol [61], and these were grouped into age stages [62]. For caprines, both tooth wear stage and age stages followed the Payne protocol [63].

### 2.3. C and N stable isotope sample preparation and analysis

Collagen extraction for carbon and nitrogen isotope analysis proceeded following established methods [64]. Whole bone fragments weighing ca. 300 mg were demineralized in 0.5M HCl solution at 5°C until demineralized (up to a week), and were then rinsed three times with deionized water until the pH became neutral. This was followed by gelatinization over 48 hours at 70°C, and then filtering and ultrafiltering using 50–90 μm EZEE^©^ filters, and previously cleaned >30 kDa Amicon^©^ ultrafilters, respectively. The purified solutions were frozen and lyophilized before being weighed into tin capsules (ca. 0.5mg of extracted collagen per tin capsule) and loaded onto the mass spectrometers.

The carbon and nitrogen isotope ratios in collagen were measured in duplicate in different runs at different days at the Isotope Facilities of the University of Cape Town (South Africa), using a Finnigan Delta plus XP continuous-flow isotope ratio mass spectrometer (Thermo Fisher Scientific, USA) after being combusted in the elemental analyzer Flash EA 1112 interfaced with it (Thermo Fisher Scientific, USA). In each run, in-house standards (valine δ^13^C = -26.8‰, δ^15^N = 12.14‰, Merck gel δ^13^C = -20.05‰, δ^15^N = 7.5‰, and seal bone δ^13^C = -11.97‰, δ^15^N = 15.84‰) were analysed in order to asses instrument precision. δ^13^C and δ^15^N mean and standard deviation values for these standards during run 1 of the samples are: Merck gel (n = 6, δ^13^C = -22.39 *±* 0.124, δ^15^N = 4.09 *±*0.042), seal bone (n = 6, δ^13^C = – 13.11 *±* 0.157, δ^15^N = 14.76 *±*0.047), Valine (n = 6, δ^13^C = -28.16 *±*0.133, δ^15^N = 11.43 *±* 0.105). Mean and standard deviation δ^13^C and δ^15^N values for these standards during run 2 of the samples are: Merck gel (n = 6 δ^13^C = -22.08 *±* 0.155, δ^15^N = 4.24 *±*0.072), seal bone (n = 6, δ^13^C = – 13.05 *±* 0.152, δ^15^N = 14.95 *±*0.064), Valine (n = 6, δ^13^C = -27.89 *±*0.159, δ^15^N = 11.71 *±* 0.075). All sample data reported was normalized to internal standards that had been calibrated against international standard materials NBS 21, IAEA N1 and N2. Stable carbon isotope ratios were expressed relative to the VPDB scale (Vienna PeeDee Belemnite) and stable nitrogen isotope ratios were measured relative to the AIR scale (atmospheric N_2_), using the delta notation (δ) in parts per thousand (‰). The reproducibility of repeated measurements of the standard materials was ≤ 0.2‰ for both δ^13^C and δ^15^N.

## 3. Results

### 3.1. Archaeozoological analysis

**Fig 2:**
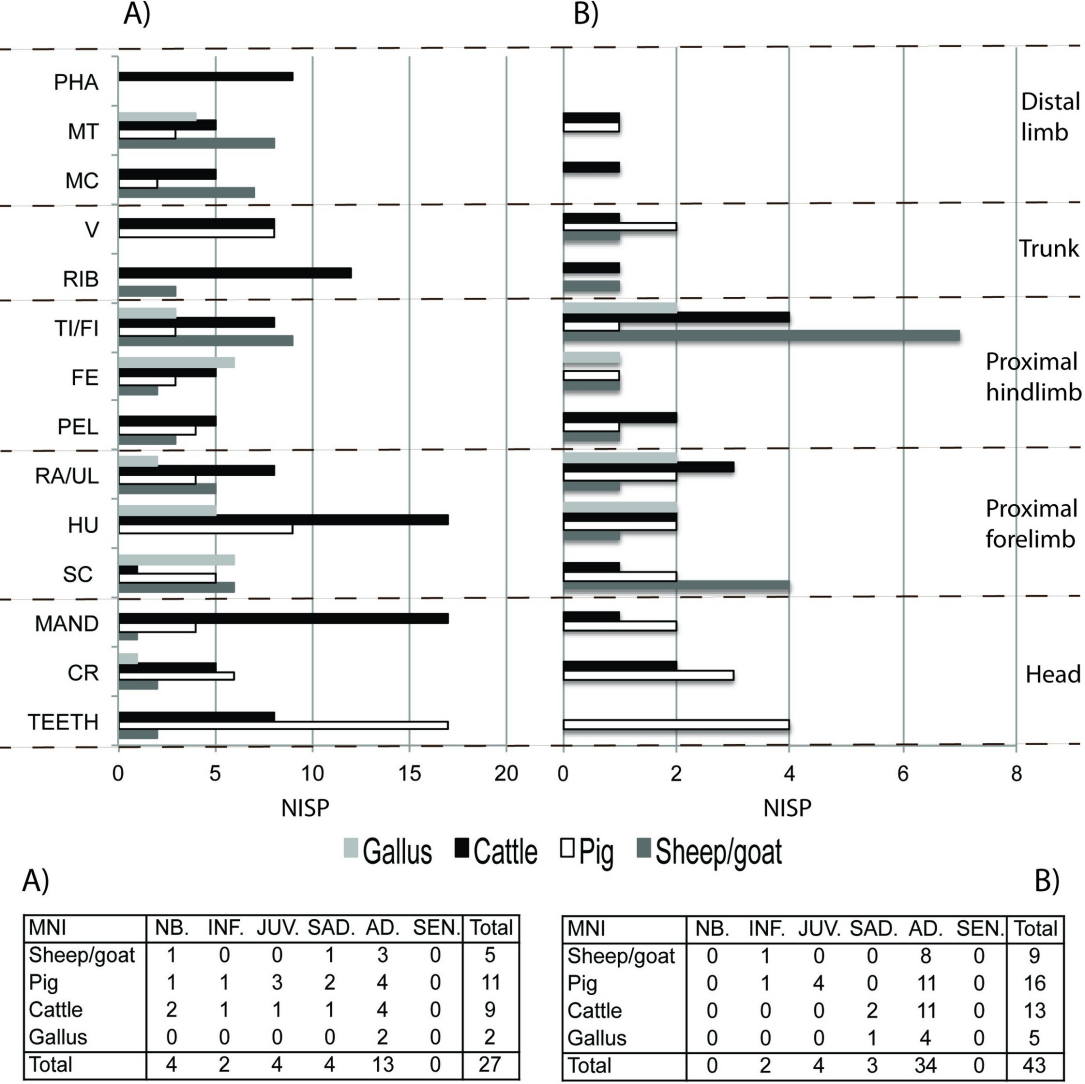
Distribution of body parts and minimum number of individuals by species on A) the occupation floor of the necropolis, and B) inside the graves of the Vila de Madrid necropolis. (Key: CR = cranium; MAND = mandible; V = vertebra; RIB = rib; SC = scapula; HU = humerus; RA/UL = radius/ulna; PEL = pelvis; FE = femur; TI/FI = tibia/fibula; MC = metacarpal; MT = metatarsal; PHA = phalanx; NB = new-born; INF = infant; JUV = juvenile; SAD = sub-adult; AD = adult: SEN = senile).

A total of 273 faunal remains indicative of funerary banquets were recovered on the occupation floor of the necropolis. The main species documented are domestic animals (97.8% of the total NISP). Among them, cattle (41.8%), pig (26.7%), sheep/goat (17.6%) and chicken (9.9%) are the main species represented. We have also documented the presence of rabbit (1.8%), fox (0.7%), red deer (0.4%), fish (0.4%) and marine mollusc (0.4%). In terms of Minimum Number of Individuals (MNI) pigs predominate (11 individuals), followed by cattle (9 individuals), caprine (5 individuals) and chickens (2 individuals) (Table A of Fig 2). Most of these individuals were sacrificed at adult ages, although newborn, infant, juvenile and sub-adult individuals were also present in the banquets performed at the necropolis (Table A of Fig 2).

Body part representation shows the presence of all parts of the skeleton, with the meat-rich upper limbs (47% of the sample) better represented than the other anatomical parts (Fig 2A). The particularly high frequency of pig teeth, cattle mandibles and cattle and pig humeri is noteworthy.

A total of 69 faunal remains were recovered from the inside of the graves as a result of funerary offerings. We documented a predominance of pig (30%) and cattle remains (27.1%), followed by caprine (24.3%) and chicken (10%). We also documented roe deer (1.4%), hare (1.4%), rabbit (1.4%) and fox (4.3%), with a similar taxonomic representation as those documented in the occupation floor of the necropolis (Fig 2B). The calculation of the MNI also shows similar results, with the presence of meat portions of at least 16 pigs, 13 cattle, 9 caprine and 5 chickens (Table B of Fig 2).

Body part representation shows the presence of all parts of the skeleton for the four main domestic animals, with the presence of elements from the axial and postcranial skeleton, including extremities (Fig 2B). The most represented part is, again, the meat-rich upper limb (67% of the sample). The particularly high frequency of caprine tibia and scapula should be highlighted. Most of these elements correspond to adult specimens, although some elements from infant, juvenile and sub-adult specimens were also present inside the graves (Table B of Fig 2).

**Fig 3:**
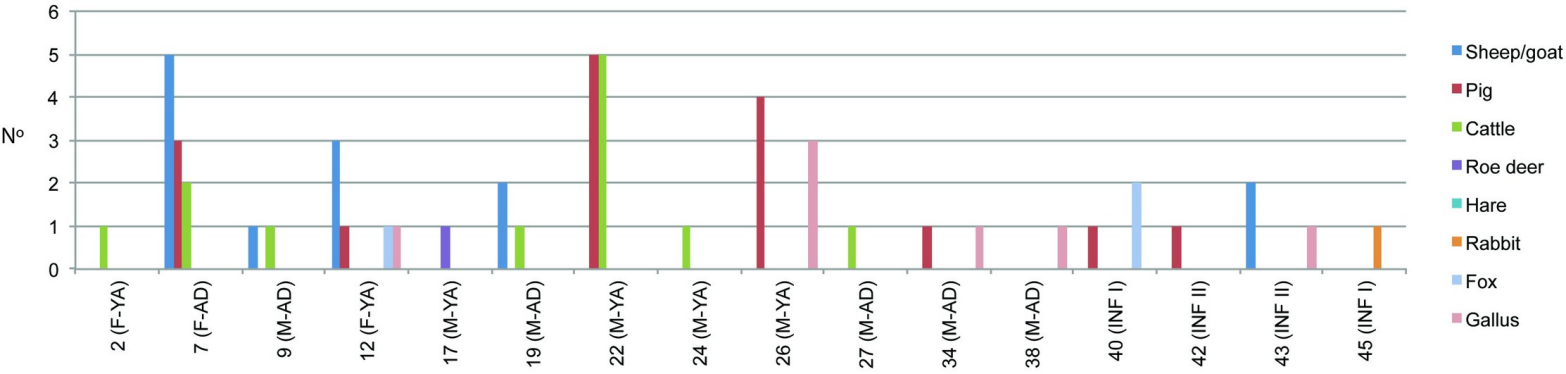
Number of meat offerings by species among the burials of the Vila de Madrid necropolis. (Key: F = female; M = male; AD = adult; YA = young adult; INF = infant).

These faunal remains were recovered in 16 inhumations in which individuals of different age and sex were buried (Fig 3). Meat offerings were not equally distributed between graves. We observe 7 graves with 1 offering, 6 graves containing between 2 and 5 offerings, and 4 graves with 6 to 10 offerings (Fig 3). Three of the 9 female burials studied (33.3%), and 9 of the 14 male burials (64.3%), present meat offerings. Of these, one female (Burial 7) has the same number of faunal remains as the male with the highest number (Burial 22). The average number of faunal samples per female burial with meat offerings is of 5.7% (17 meat offerings in 3 burials) and the average number per male burial is of 3.1% (28 meat offerings in 9 burials). The taxonomic representation also varies between graves. We documented 6 graves with the presence of 1 species, 7 graves with 2 different species, 1 grave with the presence of 3 different species, and 1 grave with 4 different species (Fig 3).

### 3.2. C and N stable isotope analysis

**Fig 4:**
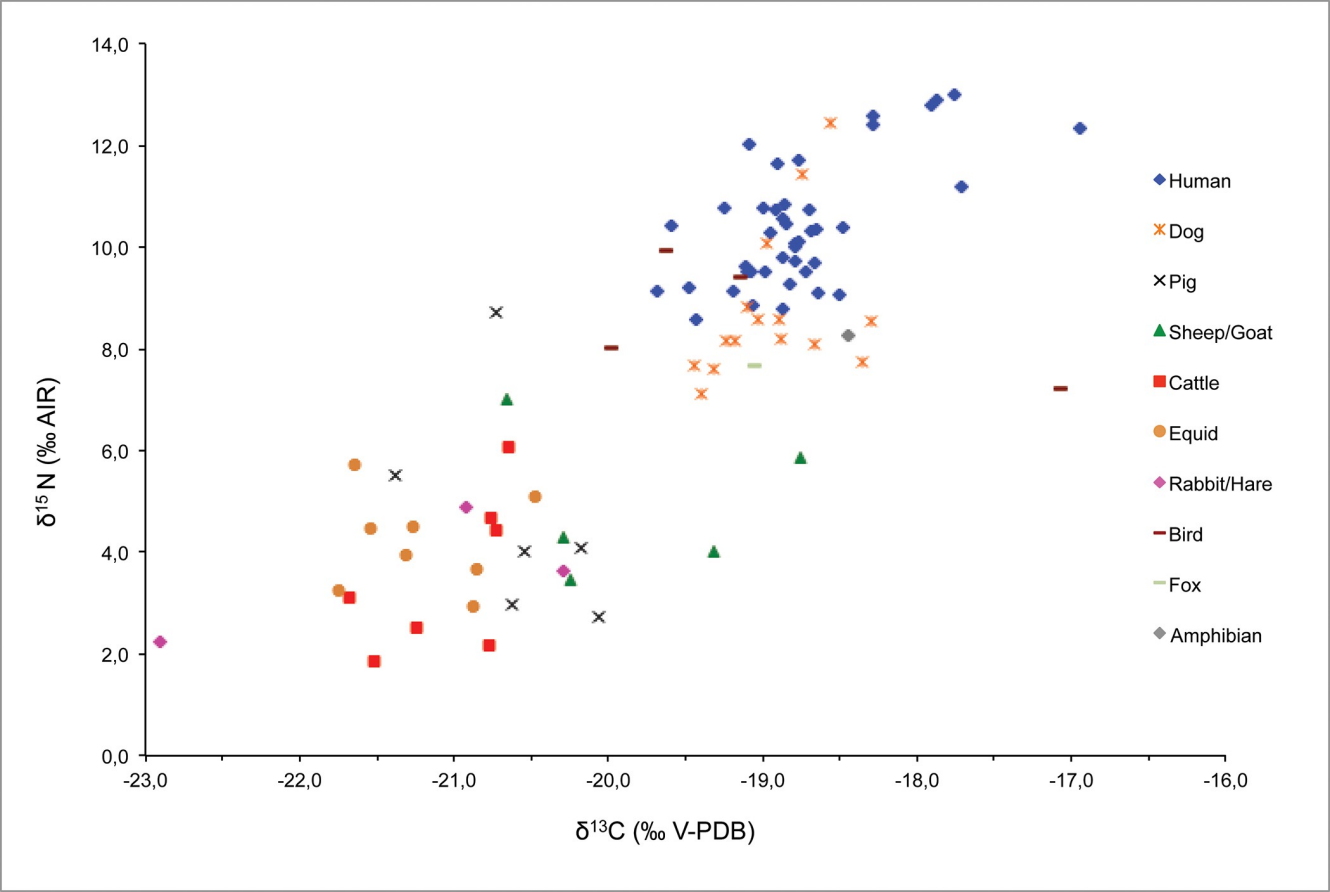
Plot of human and animal bone collagen δ^13^C and δ^15^N values from Vila de Madrid.

Samples from 41 humans and 50 faunal specimens from 11 different species (both wild and domestic) were taken for stable isotope analysis. All samples yielded sufficient collagen in the >30 kDa fraction for δ^13^C and δ^15^N analysis in duplicate, and all met published collagen quality controls following a collagen extraction protocol that includes an ultrafiltration step: appropriate CN elemental percentages together with C:N ratios between 2.9 and 3.6 [28, 29, 65]. All isotope ratio results from Vila de Madrid are shown in Tables 1 (Fauna) and 2 (Humans), and illustrated in Fig 4.

**Table 1:** Vila de Madrid δ^13^C and δ^15^N values from fauna, collagen control indicators (yield, %C, %N, C:N), S-UCT number (lab code), sampled bone, stratigraphic unit and archaeological context (oc. floor = occupation floor).

S-UCT	Species	Stratigraphic Unit	Archaeological Context	Bone	δ^13^C (‰)	δ^15^N (‰)	% Collagen	%C	%N	C:N
19095	*Equus caballus*	A771	Oc. floor	phalanx	-20.5	5.1	2.8	39.0	14.0	3.3
19096	*Equus caballus*	A597a	Oc. floor	phalanx	-20.9	2.9	3.6	24.6	8.6	3.3
19097	*Equus caballus*	A597b	Oc. floor	phalanx	-21.5	4.5	1.7	17.9	6.2	3.4
19098	*Equus caballus*	A648	Oc. floor	phalanx	-21.8	3.2	1.1	29.9	10.5	3.3
19099	*Equus caballus*	A647	Oc. floor	phalanx	-21.6	5.7	2.5	27.3	9.6	3.3
19100	*Equus caballus*	A615	Oc. floor	phalanx	-21.3	4.0	2.1	30.7	10.9	3.3
19101	*Equus caballus*	A779	Oc. floor	phalanx	-21.3	4.5	5.2	29.6	10.5	3.3
19102	*Equus caballus*	A716	Oc. floor	phalanx	-20.9	3.7	3.7	29.0	10.3	3.3
19103	*Bos taurus*	A615a	Oc. floor	astragalus	-21.2	2.5	0.8	33.0	11.6	3.3
19104	*Bos taurus*	A615b	Oc. floor	long bone	-20.8	4.7	1.4	42.3	15.2	3.2
19105	*Bos taurus*	A615c	Oc. floor	phalanx	-21.5	1.9	5.2	25.9	9.3	3.3
19106	*Bos taurus*	A647	Oc. fllor	radius	-20.8	2.2	6.6	42.2	15.2	3.2
19107	*Bos taurus*	A648	Oc. floor	femur	-21.7	3.1	0.4	39.8	13.7	3.4
19108	*Bos taurus*	A663	Grave	cubitus	-20.6	6.1	0.5	30.0	10.3	3.4
19109	*Bos taurus*	A702	Oc. floor	phalanx	-20.7	4.4	2.6	41.2	14.3	3.4
19110	*Vulpes vulpes*	A828	Grave	femur	-19.0	7.7	0.7	42.4	14.7	3.4
19111	*Gallus gallus*	A677	Grave	humerus	-19.6	9.9	3.5	42.5	15.2	3.3
19112	*Gallus gallus*	A757	Oc. floor	humerus	-19.1	9.4	0.6	37.9	12.9	3.4
19113	*Gallus gallus*	A688	Oc. floor	humerus	-20.0	8.0	0.9	30.7	10.6	3.4
19114	*Gallus gallus*	A615	Oc. floor	humerus	-17.1	7.2	3.7	42.5	15.0	3.3
19115	*Sus domesticus*	A642	Oc. floor	scapula	-20.7	8.7	1.4	42.5	14.9	3.3
19116	*Sus domesticus*	A615a	Oc. floor	scapula	-20.1	2.7	1.1	33.6	11.5	3.4
19117	*Sus domesticus*	A615b	Oc. floor	long bone	-20.2	4.1	1.5	42.2	15.2	3.2
19119	*Sus domesticus*	A647a	Oc. floor	radius	-20.6	3.0	7.5	38.4	13.7	3.3
19120	*Sus domesticus*	A647b	Oc. floor	radius	-21.4	5.5	11.4	39.8	14.1	3.3
19121	*Sus domesticus*	A681	Grave	cubitus	-20.5	4.0	1.9	36.8	13.2	3.3
19123	*Oryctolagus cuniculus*	A615	Oc. floor	pelvis	-22.9	2.2	1.9	41.3	14.8	3.2
19124	*Oryctolagus cuniculus*	A647	Oc. floor	humerus	-20.9	4.9	3.5	42.4	14.7	3.4
19125	*Lepus granatensis*	A696	Grave	humerus	-20.3	3.6	7.6	42.5	15.4	3.2
19126	Amphibian	A 615	Oc. floor	femur	-18.4	8.3	0.8	39.6	14.1	3.3
19128	*Capra hircus*	A615	Oc. floor	radius	-20.2	3.5	2.1	42.1	15.2	3.2
19129	*Ovis aries*	A812	Well	vertebra	-19.3	4.0	0.9	41.2	14.5	3.3
19130	*Ovis aries*	A642	Oc. floor	phalanx	-20.7	7.0	2.6	40.7	14.6	3.2
19131	*Ovis aries*	A647	Oc. floor	radius	-18.7	5.9	9.8	41.8	15.1	3.2
19132	*Ovis aries*	A653	Oc. floor	radius	-20.3	4.3	9.6	34.2	12.3	3.2
19133	*Canis familiaris*	A597	Oc. floor	vertebra	-19.4	7.7	5.8	22.6	8.2	3.2
19134	*Canis familiaris*	A648	Oc. floor	femur	-18.6	12.5	2.7	35.6	12.4	3.3
19135	*Canis familiaris*	A615	Oc. floor	vertebra	-18.7	8.1	0.7	40.6	14.5	3.3
19136	*Canis familiaris*	A686	Oc. floor	vertebra	-19.3	7.6	1.4	33.1	11.7	3.3
19137	*Canis familiaris*	A688	Oc. floor	vertebra	-18.9	8.6	0.9	36.9	13.1	3.3
19138	*Canis familiaris*	A713	Oc. floor	vertebra	-18.9	8.2	1.6	42.1	15.2	3.2
19139	*Canis familiaris*	A716	Oc. floor	vertebra	-18.3	8.5	0.8	41.1	14.7	3.3
19140	*Canis familiaris*	A752	Oc. floor	vertebra	-18.4	7.7	1.9	41.2	15.0	3.2
19141	*Canis familiaris*	A757	Oc. floor	vertebra	-19.4	7.1	0.5	41.4	14.5	3.3
19142	*Canis familiaris*	A776	Well	vertebra	-19.0	10.1	2.1	36.3	13.0	3.3
19143	*Canis familiaris*	A789	Well	vertebra	-19.1	8.8	2.3	34.1	12.1	3.3
19144	*Canis familiaris*	A807/2	Oc. floor	vertebra	-19.2	8.2	1.1	34.5	11.9	3.4
19145	*Canis familiaris*	A809	Oc. floor	vertebra	-18.7	11.4	5.1	32.5	11.6	3.3
19146	*Canis familiaris*	A812	Well	vertebra	-19.0	8.6	2.2	25.3	9.2	3.2
19147	*Canis familiaris*	A814	Well	vertebra	-19.2	8.2	2.9	29.0	10.5	3.2

**Table 2:** Vila de Madrid δ^13^C and δ^15^N values from humans, collagen control indicators (yield, %C, %N, C:N), S-UCT number (lab code), burial number, type of burial, stratigraphic unit, number of offerings, number of species present as offerings, sex and age category.

S-UCT	Burial	Type of burial	Stratigraphic Unit	N° of meat offerings	N° of species offered	Sex	Age Category	δ^13^C (‰)	δ^15^N (‰)	% Coll.	%C	%N	C:N
19176	2	Simple pit	A634	1	1	Female	Young adult	-19.1	9.5	0.9	35.0	12.3	3.3
19177	4	Simple pit	A640	0	0	Indet.	Newborn	-18.7	10.8	0.7	29.2	10.1	3.4
19178	5	Tegulae coffin	A657	0	0	Indet.	Infant I	-18.3	12.4	1.6	39.4	14.2	3.2
19179	6	Simple pit	A660	0	0	Indet.	Newborn	-18.8	10.5	0.9	33.8	11.7	3.4
19180	8	Wooden coffin	A672	0	0	Indet.	Infant I	-18.5	9.1	0.7	39.0	14.0	3.3
19181	9	Simple pit	A675	2	2	Male	Adult	-18.7	10.3	1.0	40.2	14.5	3.2
19182	33	Wooden coffin	A803/678/677	0	0	Female	Adult	-18.8	9.3	3.5	42.4	15.4	3.2
19183	7	Unknown	A681	10	3	Female	Adult	-18.8	10.1	2.8	42.0	15.3	3.2
19184	10	Simple pit	A693	0	0	Indet.	Newborn	-18.8	11.7	1.0	30.7	10.7	3.4
19185	3	Simple pit	A636	0	0	Female	Young adult	-19.0	9.5	0.8	39.3	13.7	3.4
19186	12	Simple pit	A717	6	4	Female	Young adult	-19.1	8.8	0.8	38.3	13.7	3.3
19187	13	Simple pit	A719	0	0	Indet.	Newborn	-17.9	12.8	0.7	31.9	11.3	3.3
19188	16	Simple pit	A737	0	0	Indet.	Infant II	-19.6	10.4	0.3	40.1	13.7	3.4
19189	17	Simple pit	A742	1	1	Male	Young adult	-18.8	10.1	0.6	35.7	12.7	3.3
19190	19	Simple pit	A751	3	2	Male	Adult	-18.9	10.3	3.0	41.9	15.2	3.2
19191	20	Oval simple pit	A758	0	0	Male	Adult	-18.9	10.7	4.3	39.2	13.7	3.3
19192	21	Simple pit	A759	0	0	Indet.	Infant II	-19.2	9.1	0.7	40.0	14.3	3.3
19193	22	Tegulae coffin	A766a	10	2	Male	Young adult	-18.5	10.4	1.2	41.9	15.1	3.2
19195	23	Simple pit	A769	0	0	Indet.	Infant I	-17.8	13.0	3.2	42.1	15.3	3.2
19196	24	Simple pit	A772	1	1	Male	Young adult	-18.9	8.8	2.9	42.3	15.4	3.2
19197	25	Simple pit	A775	0	0	Female	Adult	-18.9	9.8	2.7	42.6	15.4	3.2
19198	26	Simple pit	A780	7	2	Male	Young adult	-18.8	10.0	1.4	31.7	11.5	3.2
19199	28	Simple pit	A786	0	0	Female	Adult	-18.8	9.7	0.9	39.8	14.3	3.3
19200	29	Simple pit	A792	0	0	Male	Adult	-18.6	9.1	0.9	38.2	13.6	3.3
19201	32	Unknown	A801-802	0	0	Male	Adult	-18.9	10.6	0.5	34.1	12.0	3.3
19202	34	Simple pit	A806	2	2	Male	Adult	-18.7	9.7	3.0	38.2	13.9	3.2
19203	35	Simple pit	A810	0	0	Male	Adult	-19.5	9.2	0.7	37.0	13.0	3.3
19204	37	Wooden coffin	A820	0	0	Female	Adult	-18.7	9.5	1.9	41.6	14.9	3.2
19205	38	Tegulae coffin	A822	1	1	Male	Adult	-17.7	11.2	2.3	42.5	15.5	3.2
19206	39	Simple pit	A825	0	0	Indet.	Newborn	-16.9	12.4	0.8	34.5	12.3	3.3
19207	40	Wooden coffin	A829	3	2	Indet.	Infant I	-19.0	10.8	2.1	35.7	12.3	3.4
19211	41	Wooden coffin	A832	0	0	Indet.	Infant I	-18.3	12.6	0.6	39.4	14.0	3.3
19212	42	Simple pit	A836	1	1	Indet.	Infant II	-19.4	8.6	0.7	39.8	14.0	3.3
19213	43	Wooden coffin	A839	3	2	Indet.	Infant II	-19.7	9.1	0.7	35.9	12.5	3.4
19214	44	Simple pit	A842	0	0	Indet.	Infant I	-18.9	11.6	1.3	28.5	10.2	3.3
19215	45	Simple pit	A844	1	1	Indet.	Infant I	-17.9	12.9	3.5	39.1	13.9	3.3
19216	46	Simple pit	A847	0	0	Indet.	Newborn	-19.1	12.0	1.5	32.2	11.1	3.4
19217	47	Simple pit	A850	0	0	Indet.	Newborn	-19.1	9.5	1.6	22.3	8.0	3.3
19218	27	Simple pit	A9004	1	1	Male	Adult	-19.2	10.8	2.3	38.9	13.5	3.4
19219	56	Simple pit	A9013	0	0	Male	Young adult	-18.9	10.8	3.5	39.4	14.2	3.2
19220	57	Simple pit	A9015	0	0	Female	Adult	-19.1	9.6	4.0	39.4	14.2	3.2

Analysing the faunal carbon values, it can be seen that the terrestrial herbivore δ^13^C mean value is -20.9 ± 0.9 (1σ) ‰ and its minimum and maximum values are -22.9‰ and -18.7‰ respectively. Other than the herbivore (*Oryctolagus cuniculus*) at the lowest value and the herbivore (*Ovis aries*) at the highest value, all other herbivores group between -21.8‰ and -19.3‰, which is generally compatible with typical C_3_ terrestrial ecosystems. The mean carbon isotope composition of the omnivores analyzed is -19.3 ± 0.9 (1σ) ‰, and its minimum and maximum values are -21.4‰ and -17.1‰ respectively. By removing the highest (*Gallus gallus*) and lowest (*Sus domesticus*) values, all other omnivores group between -20.7‰ and 18.3‰. These and the single carnivore value analyzed (-19.0‰) are consistent with the general herbivore values and a terrestrial C_3_ food ecosystem respectively.

Analysing the faunal nitrogen values, the herbivore mean δ^15^N value is 4.1 ± 1.3 (1σ) ‰ and has minimum and maximum values of 1.9 and 7.0‰ respectively, with a homogeneous dispersal of all species between this interval, and gives the background for the herbivore trophic level at the site. The mean δ^15^N value of the omnivores analysed is 7.8 ± 2.4 (1σ) ‰, with minimum and maximum values of 2.7 and 12.5‰, respectively. Although the overall mean value of the omnivores seems to be situated almost a trophic level above that of the herbivores, when analysed at the species level we observe different patterns. While *Sus domesticus* (4.7 ± 2.2 [1σ] ‰) have values similar to those of herbivores, *Canis familiaris* (8.8 ± 1.5 [1σ] ‰) and *Gallus gallus* (8.6 ± 1.2 [1σ] ‰) are clearly situated at a higher trophic level than the herbivores, suggesting different feeding patterns and social interaction of these species with humans. The mean δ^15^N value of the one wild carnivore analysed (*Vulpes vulpes*) is 7.7‰, which is 3.6‰ higher than the herbivore mean value and situates the carnivore almost one complete step higher than the herbivores in the food chain. However, the carnivore value is a bit lower compared to that of *Canis familiaris* and *Gallus gallus* mean values, which are similar to those from humans, suggesting a close link to humans in the domestic area that might be linked to being fed or scavenging human food refuse. Unfortunately, other than that of an unidentified amphibian value, no aquatic resources were available for sampling at this site, and thus the marine and freshwater specific ‘baseline’ is lacking for Vila de Madrid.

When analysed altogether (n = 41), humans from Vila de Madrid have δ^13^C and δ^15^N mean values of −18.8 ± 0.5 (1σ) ‰ (min: −19.7 ‰, max: −16.9 ‰) and 10.4 ± 1.2 (1σ) ‰ (min: 8.6‰, max: 13.0‰), respectively. When excluding infant individuals to avoid potential interference from breastfeeding (n = 23), humans from Vila de Madrid have δ^13^C and δ^15^N mean values of −18.8 ± 0.3 (1σ) ‰ (min: −19.5 ‰, max: −17.7 ‰) and 9.9 ± 0.7 (1σ) ‰ (min: 8.8‰, max: 11.2‰), respectively. These values show that diet was based on terrestrial C_3_ resources. They also suggest that humans were placed in a higher trophic level than herbivores and slightly higher than both domestic omnivores and the wild carnivore analysed (Fig 4).

When grouped by sexes, females (n = 9) have δ^13^C and δ^15^N mean values of −18.9 ± 0.2 (1σ) ‰ (min: −19.1‰, max: −18.7‰) and 9.5 ± 0.4 (1σ) ‰ (min: 8.8‰, max: 10.1‰) respectively, while males (n = 14) show δ^13^C and δ^15^N mean values of −18.8 ± 0.4 (1σ) ‰ (min: −19.5‰, max: −17.7‰) and 10.1 ± 0.7 (1σ) ‰ (min: 8.8‰, max: 11.2‰) respectively. When grouped by age categories, the Newborn-Infant I age category (n = 14) have δ^13^C and δ^15^N mean values of −18.4 ± 0.6 (1σ) ‰ (min: −19.1‰, max: −16.9‰) and 11.6 ± 1.3 (1σ) ‰ (min: 9.1‰, max: 13.0‰) respectively, individuals from the Infant II category (n = 4) show δ^13^C and δ^15^N mean values of −19.5 ± 0.2 (1σ) ‰ (min: −19.7‰, max: −19.2‰) and 9.3 ± 0.8 (1σ) ‰ (min: 8.6‰, max: 10.4‰) respectively, Young Adults (n = 8) have δ^13^C and δ^15^N mean values of −18.9 ± 0.2 (1σ) ‰ (min: −18.5‰, max: −19.1‰) and 9.7 ± 0.7 (1σ) ‰ (min: 8.8‰, max: 10.8‰) respectively, and Adults (n = 15) show δ^13^C and δ^15^N mean values of −18.8 ± 0.4 (1σ) ‰ (min: −17.7‰, max: −19.5‰) and 10.0 ± 0.6 (1σ) ‰ (min: 9.1‰, max: 11.2‰) respectively.

**Fig 5:**
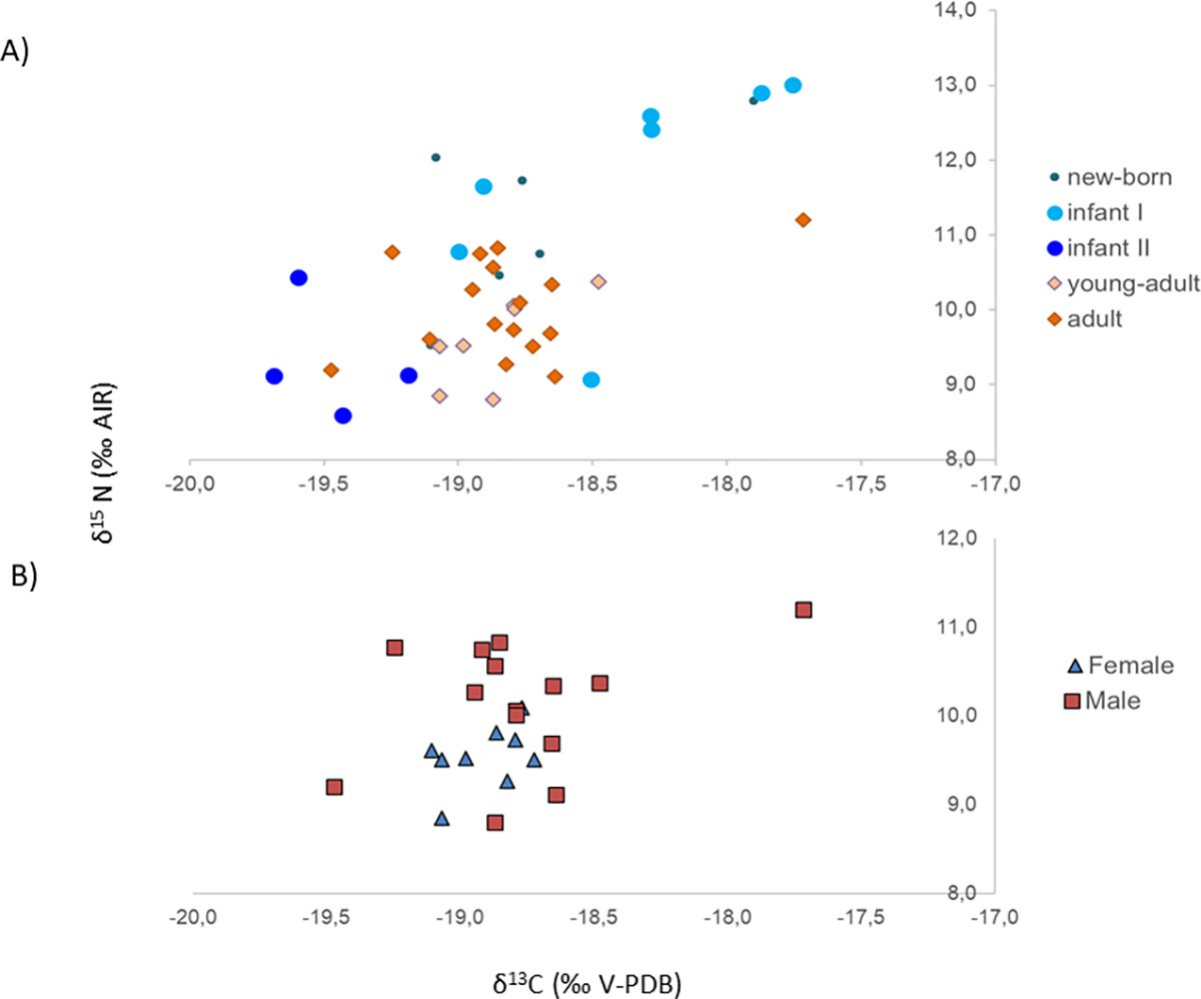
Plot of human bone collagen δ^13^C and δ^15^N values from Vila de Madrid sorted by (A) age groups and (B) sex -only from young adults and adults-.

Overall, and with exceptions that will be further discussed, the adult and young adult individuals portray a homogeneous protein dietary input regardless of age (Fig 5A) (δ^13^C: Shapiro-Wilk test, p = 0.002, Mann-Whitney U test, p = 0.491; δ^15^N: Shapiro-Wilk Test, p = 0.327; Levene’s Test, p = 0.738; Student’s t, p = 0.391), and only slight differences depending on biological sex (Fig 5B), being statistically significant only in δ^15^N values (δ^13^C: Shapiro-Wilk test, p = 0.002, Mann-Whitney U test, p = 0.306; δ^15^N: Shapiro-Wilk Test, p = 0.310; Levene’s Test, p = 0.042; Welch’s t test, p = 0.013).

**Fig 6:**
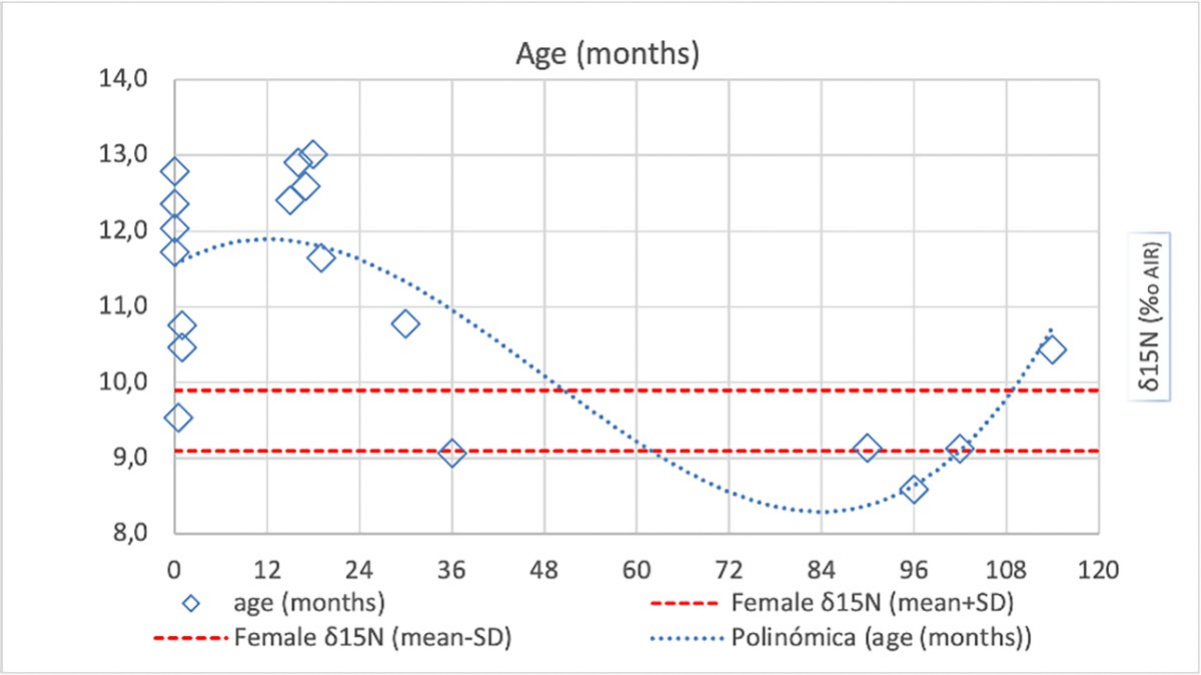
Plot of human bone collagen δ^15^N values from Vila de Madrid pre-adults plotted against age in months.

When assessing isotopic values grouped in the early-life age stages (newborn, Infantile I, Infantile II), several interesting patterns can be observed (Fig 5A). Other than the age group differences, it is also interesting to see if breastfeeding and weaning practices amongst this population can be detected. Looking at Fig 6, it can be observed how overall the δ^15^N values are higher than that of female adults until ca. 2 years of age, when they start to drop until reaching the mean female adult value. This would suggest that breastfeeding was common until the age of 2, the moment at which the isotopic impact of weaning starts to become visible, reaching a regular diet possibly from 3 years of age onwards. The newborn individuals are special cases, as those who died immediately after birth would not have had time to show the increase in trophic level obtained from breastfeeding in their collagen isotopic signature, which requires at least a few months post-birth (but still being visible before 1 year of age).

**Fig 7:**
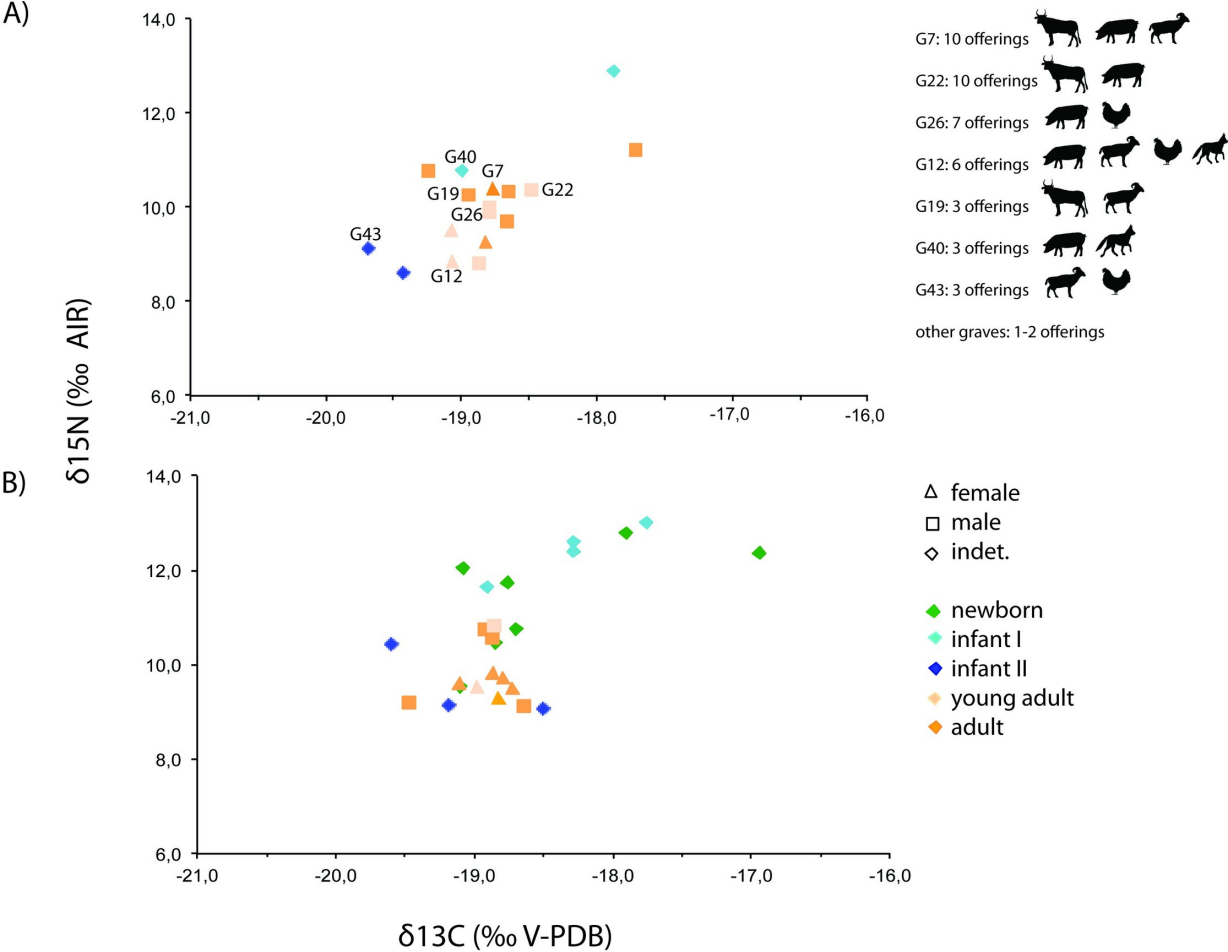
Plot of human bone collagen δ^13^C and δ^15^N values from Vila de Madrid according to the (A) presence and (B) absence of meat offerings and their number, and sex and age of the individuals.

Finally, in order to shed more light on funerary meals at the Vila de Madrid necropolis, human isotopic data and information on offerings, sex and age of the individuals have been plotted together (Fig 7). No apparent relationship between differences in isotope values and the presence/absence of offerings in the burial nor the number of offerings, is observed. Individuals buried with a high number of offerings (Burials 7, 12, 22 and 26) show isotopic values similar to those of other burials without offerings. Similarly, there is no relationship between the type of offering (species, portions, etc.) and the sex of the human individuals. However, males show a slight higher number of meat offerings than females, as well as higher δ^15^N values, although these values might be impacted by the smaller number of females in the sample. There is also no clear relationship between a specific age and specific isotope values; the only significant age trait is that no newborn burials possessed meat offerings. In this sense, despite the infantile group (newborn, infant I, infant II) having a high number of individuals (18 individuals), only 4 of them have meat offerings.

## 4. Discussion

First of all, we have to point out that very few offerings were recovered in the collective funerary structure of Vila de Madrid. Meat offerings were only recovered in 16 graves of a total of 59. This fact seems to indicate that not all of the deceased received meat offerings, and nor did their families perform banquets, despite the fact that these rituals were stipulated by law according to written sources (Pliny, Epist. IV, 2; Cicero, De leg.II, 22, 55–57; Tacitus, Ann.VI, 5). Having pointed this out, this study has demonstrated that mainly terrestrial domestic animals were consumed at the Vila de Madrid funerary meal rituals and banquets. Wild animals, as well as marine and freshwater resources, were scarce or not present at all. Specifically, the most consumed meat during the funerary banquets and used for the offerings to the deceased were those of pork and beef, followed by caprines and chicken. This pattern is observed widely throughout the western range of the ancient Roman Empire, as for example at the necropolis of Can Bel at Pineda de Mar (Catalonia) [9], at Rodez at Aveyron (France) [66] and at Sagnes à Pontarion at Creuse (France) [67].

Furthermore, this study shows that the most consumed portions at Vila de Madrid funerary rituals and among the offerings to the deceased were the proximal elements of the limbs (scapula, humerus, radius, ulna, pelvis, femur, tibia), although other portions are also present. This representation suggests that there may have been a preference or a selection for offering and consuming meat-rich portions. These portions, however, came from mainly adult individuals, which shows that older animals past their peak reproductive or commercial value were selected in these rituals. This is an important point, as it suggests that only animals that could not be exploited for other purposes were sacrificed and therefore the economic burden of sacrifices could be minimized. It might also suggest that a special dedicated purchase of tender meat was not customary for these occasions, regardless of their importance. Therefore, the archaeozoological study presented here adds more evidence to the interpretation made of this structure, as a *collegium funeraticium*, as we have documented that meat offerings and banquets were not as typical as written sources may indicate (Pliny, Epist. IV, 2; Cicero, De leg.II, 22, 55–57; Tacitus, Ann.VI, 5; Petronius, Satyrion 65), and when they were performed, the best cuts of tender meat were generally not used. We note, however, that portions that did not contain bones (e.g. viscera, testicles, breasts) could have also been consumed in these rituals, or even that attendees could have taken portions away, and both scenarios would result in no traces of them in the archaeological record.

Isotopic data show that overall, the diet of the humans interred at Vila de Madrid was based on terrestrial C_3_ resources, and likely included plants as a main staple. However, that human isotope values are one trophic level higher than those of herbivores and some omnivores (pigs) suggests that meat from these animals were also consumed on a regular basis for the population as a whole. Additionally, there is no clear isotopic signal that would suggest regular consumption of other domestic omnivores such as chicken and dog, who show similar isotopic values to those of humans. Similarly, there is no isotopic evidence of the consumption of wild carnivores or fish. Of course, this cannot determine that such resources were never consumed, but rather it indicates that regular feeding on these types of animals did not occur.

Interestingly, while the main species present at the funerary rituals, including pig, cattle and sheep/goat, seem to also have been consumed regularly during life, the other common funerary ritual species at Vila de Madrid, chicken, was not. If this was the case, isotopic results would support the archaeozoological data that show chicken remains in Roman domestic contexts (in Hispania but also elsewhere in the Roman Empire) were present despite not being a common species [68–70]. The presence of domestic fowl has also been documented in other necropolis, being the second most frequently occurring animal in human graves in Britain and France after that of pigs [71]. This large presence of chicken in cemeteries has been related to the consumption or offering of a luxurious food [68]. It should be also noted that this bird had a remarkable symbolic value during Antiquity, especially linked to the cock [72], being also the emblem of Attis, the solar god of resurrection [73]. In Vila de Madrid, although it is not the second most abundant species, the presence of chicken is remarkable because it shows the use of a luxury food, or at least of an uncommon meat, by some relatives that performed rituals in the necropolis.

Another aspect to highlight is the presence of wild animals in the funerary meals and meat offerings at Vila de Madrid necropolis. We have documented the presence of one ulna and one humerus of a fox, and a fragment of a red deer metacarpus in the occupation floor of the necropolis. We have also documented the presence of one fragment of roe deer metatarsus (Burial 17), one fragment of fox humerus (Burial 12), and a fox femur and vertebra (Burial 40) inside the graves. The presence of wild animals in funerary contexts, although uncommon, is not rare. Some examples include: the necropolis of Colombes in Beaucaire (France), where wild boar, red deer and hare remains were documented [74]; the Eastern Cemetery of London, (England) with the presence of hare, red deer, roe deer and pine marten remains [12]; the necropolis of Can Trullàs (Catalonia), with the presence of fox, wild boar, and red deer remains [75]; or the temples of Bancroft and Chelmsford (England), where faunal remains of fox, hare, roe deer and red deer were recovered [76]. The authors of these studies suggested that the presence of these rare animals could be linked with symbolic offerings or, conversely, it could be a way to avoid slaughtering domestic animals and instead use wild mammals as a kind of surrogate victim.

Another interesting aspect about the human sample set is the presence of a large number of infantile individuals at Vila de Madrid necropolis, allowing to explore non-adult dietary patterns. The age groups “Newborn” and “Infantil I” are more heterogeneous, and the age group “Infantile II” is a bit different. For both “Newborn” and “Infantile I” groups, while some of their individuals group together with the other age groups, a considerable amount of them stand out by having higher δ^13^C and δ^15^N values than the other groups. Knowing that breastfeeding places babies one trophic step higher than their mothers [77], then higher values suggest that such individuals were still breastfeeding when they died. The other individuals from this age group, however, show no clear isotopic signal of breastfeeding, suggesting that they had already been weaned or were never breastfed at the time of their death. Regarding age group “Infantile II”, their values are slightly lower in δ^13^C and δ^15^N than the age groups “Young Adult” and “Adult” suggesting that children already of a certain age might have had a different protein input proportion in their diet than that of older individuals. This might be in line with Roman children assuming a more restricted diet compared to male diet [78], but could also reflect the “no-survivor” population segment of this age group (untimely deaths of children could result from nutritional deficiencies or sub-optimum nutrition).

Regarding possible dietary differences due to biological sex, it is interesting to see that many of the male individuals show higher δ^15^N values than the females even if showing similar δ^13^C values. This slight difference in dietary protein input between sexes could be due to several reasons. For one, it could mean that males ate more protein-rich foods (i.e. meat) than females. It could also show that males ate more freshwater fish than females, although as there are no remains of freshwater resources available as reference from the site, this cannot be confirmed. Assuming that males ate more meat than females, this could mean or that social-cultural tastes for foods were different between sexes, or that more males than females had access to protein-rich resources due to perhaps custom, social status, wealth or medical recommendations [79]. In this respect, there is evidence from literary sources that males and females were advised to eat different types of food based on the ‘humors’. Males were hot and dry so they were to eat cold and wet foods (e.g. fish). Females were thought to be cold and wet, so they were to eat hot and dry foods (e.g. cereals) [79].

In this regard, the adult male buried in Burial 38 must be highlighted. He shows a clearly higher δ^13^C value and a slightly higher δ^15^N value when compared to the other individuals, suggesting that he consumed possibly a combination of C_4_ resources and aquatic protein that shifted him more in the δ^13^C than δ^15^N value, enough to be reflected in the collagen signal even if the diet was still based on C_3_ terrestrial resources. Curiously, this individual is of an older age at death (older than 45yo), and his burial is one of the few associated with fowl remains and buried in a tegulae box present in the necropolis. The other tegulae burial analysed (Burial 22, with a young adult male accompanied by10 meat offerings) has lower δ^13^C and δ^15^N values than the individual from Burial 38, but still higher than that of the other adult individuals. Future residential mobility isotope analysis at Vila de Madrid will shed light about the possibility that some of these male individuals could have been non-local.

## 5. Conclusions

The archaeozoological and anthropological study presented here has allowed us to investigate the funerary offerings and banquets developed in the Vila de Madrid necropolis, and the main types of protein resources that the deceased consumed during life. This interdisciplinary approach has exposed three important points. Firstly, such funerary rituals were not common in the collective funerary structure of the necropolis. Secondly, the most commonly used meats in the rituals–pork, beef, and caprine–were the most consumed meats in life. Finally, these meats were mainly consumed from adult individuals. These data suggest that despite offerings and banquets being stipulated by law, not everyone could afford to make large or rich offerings. This is likely the case of the humans buried at Vila de Madrid. At the same time, however, the presence of fowl offerings and the presence of meat-rich portions suggest that the relatives of the deceased tried to follow the law as far as possible. On the other hand, we have also documented a high consumption of meat of adult males during their lives but only a slightly higher presence of offerings in adult male burials. These ritual and diet differences likely show sex inequalities during life that were extrapolated to the funerary rituals.
